# Authors' Reply

**DOI:** 10.18502/jovr.v17i4.12346

**Published:** 2022-11-29

**Authors:** Ramesh Venkatesh, Kushagra Jain, Arpitha Pereira, MB Thirumalesh, Naresh Kumar Yadav

**Affiliations:** ^1^Department of Retina and Vitreous, Narayana Nethralaya, Bangalore, India; ^3^https://orcid.org/0000-0002-4479-9390

##  Dear Editor,

We thank the author for showing interest in our article titled “Torpedo Retinopathy” and for providing important comments on it.^[1]^ Classically, torpedo maculopathy is considered as a retinal pigment epithelium (RPE) defect, typically in the temporal macula with a characteristic pointed, "torpedo" shape, with one tip pointing toward the macula. Several theories have been floating as to the etiology, including an hypopigmented RPE nevus, a developmental defect in the "fetal temporal bulge", or failure of the RPE to close overlying the region near the emissary canal of the long posterior ciliary artery and nerve.^[2,3,4]^ Our paper describes cases of torpedo lesions located at sites other than the macula in the fundus.^[1]^ Hence, we titled the paper as “Torpedo Retinopathy” and suggested a change in the routinely used nomenclature of “Torpedo Maculopathy”. With the use of advanced retinal imaging techniques such as optical coherence tomography (OCT), OCT-angiography, multicolor imaging, and adaptive optics imaging, it is increasingly becoming clear that the inner choroid, mainly the choriocapillaris layer gets affected in the torpedo lesions.^[5,6,7]^ Hence, we do back the suggestion by the author that the revised nomenclature for the torpedo lesions in the fundus be termed as “Torpedo Chorioretinopathy” as it addresses the issues both related to its location and its involvement of the retina and choroid.
